# Two decades of sperm sex-sorting in animals: A comprehensive systematic review and meta-analysis of techniques, effectiveness, and global research trends (2005–2025)

**DOI:** 10.14202/vetworld.2025.4009-4024

**Published:** 2025-12-23

**Authors:** Thatawat Yodrug, Orachun Hayakijkosol, Tuempong Wongtawan

**Affiliations:** 1 Animal Innovation Research Group, Akkhraratchakumari Veterinary College, Walailak University, Nakhon Si Thammarat 80160, Thailand; 2 College of Science and Engineering, Academy Division, James Cook University, Queensland, Australia; 3 Centre for One Health, Walailak University, Nakhon Si Thammarat 80160, Thailand

**Keywords:** assisted reproduction, centrifugation, flow cytometry, livestock production, magnetic beads, microfluidics, offspring sex-ratio, One Health, pregnancy rate, sperm sex-sorting

## Abstract

**Background and Aim:**

Sperm sex-sorting technologies are crucial tools for enhancing reproductive management, production efficiency, sustainability, and animal welfare. Although widely used across various species, the effectiveness of these technologies, particularly between flow cytometry and emerging alternative methods, remains inconsistently reported. This systematic review and meta-analysis examined research published worldwide over the past 20 years to compare the performance of different sperm sex-sorting techniques, evaluate pregnancy outcomes and offspring sex ratios, and identify research trends, limitations, and knowledge gaps.

**Materials and Methods:**

A thorough search of PubMed, Scopus, and Google Scholar identified studies published from 2005 to 2025. Ninety-one studies met the criteria for systematic review, and 22 were included in the quantitative synthesis. Data extraction included species, sex-sorting method, experimental design, sperm quality parameters, pregnancy outcomes, and female-offspring proportions. Random-effects proportional meta-analyses were conducted for pregnancy rate and sex-ratio outcomes. Heterogeneity was assessed using I², Tau², and H², while publication bias was evaluated with funnel plots and Egger’s/Begg’s tests.

**Results:**

Flow cytometry was the most widely used method (64 studies), especially in North America, while research in Asia focused more on alternative techniques such as antibody-based sorting, centrifugation, modified buffers, magnetic beads, and microfluidics. Meta-analysis of *in vivo* studies showed an overall pregnancy rate of 46.47% for flow cytometry, with significant differences among cattle, buffalo, and deer. Centrifugation-based methods had a higher pooled pregnancy rate of 66.49%, though this was based on only three studies. The overall proportion of female offspring after flow-sorting across all species was 81.72%, with cattle reaching 84.40%. Significant heterogeneity was present (I² > 73%), but no publication bias was found.

**Conclusion:**

Flow cytometry remains the global gold standard for sperm sex-sorting, offering high accuracy, but it is limited by cost, technical requirements, and reduced accessibility in developing regions. Growing research activity in Asia highlights increasing interest in alternative, low-cost methods; however, *in vivo* validation remains limited. Future efforts should focus on large-scale field trials, standardized protocols, comparative studies with unsorted semen, and expanded research across species, including wildlife, to improve practical applicability, sustainability, and One Health benefits.

## INTRODUCTION

Sperm sex-sorting technology has become a valuable tool for enhancing livestock productivity, economic efficiency, animal welfare, food security, and overall sustainability [1, 2]. By allowing breeders to predetermine the sex of offspring, this technology supports strategic breeding goals, such as producing female calves for dairy or male progeny for meat production [3]. In dairy systems, female-biased sexing significantly reduces the number of unwanted male calves, addressing ethical concerns and improving dam welfare [4]. Moreover, sex-sorting boosts production efficiency and environmental sustainability by reducing the need for replacement animals, thereby lowering resource use, including feed, water, and land, and reducing greenhouse gas emissions [5]. When used to propagate high-genetic-merit females, it accelerates genetic progress and enhances productivity and feed efficiency [6]. Overall, these benefits strengthen both the economic and ethical sustainability of modern livestock systems.

The production of sexed semen depends on the differential presence of X- or Y-bearing spermatozoa, allowing the creation of offspring of the desired sex [7]. Several sorting methods have been developed to exploit the inherent differences between X- and Y-sperm. X-bearing sperm contain about 3.8% more DNA than Y-bearing sperm, which provides the biological basis for their separation [8]. Techniques such as flow cytometry, albumin sedimentation, and Percoll density-gradient centrifugation use these DNA-content differences [1]. Among these, flow cytometry, especially the Beltsville sperm-sorting system, remains the most effective method for *in vitro* separation and for increasing the number of female offspring in practical applications [9–11]. Its use has been extensively validated across various species, especially ruminants [12–17]. However, flow cytometry has several limitations, including sperm damage, high operational costs, technical complexity, and relatively lower pregnancy rates [8].

Centrifugation-based methods, especially those using albumin gradients, are alternative strategies that separate sperm by density, motility, or surface charge [18]. The bovine serum albumin gradient method is straightforward and well-established [19, 20], providing the benefit of preserving acrosomal integrity and maintaining sperm yield. However, its overall effectiveness varies across different studies [20].

Immunological sorting offers another promising, non-invasive, and cost-effective alternative to flow cytometry [21, 22]. This method uses antibodies that selectively bind to sperm membrane antigens, reducing the motility or viability of specific sperm populations [23, 24]. Immunological techniques generally produce higher sperm counts per dose [18] and are suitable for large-scale commercial use [21]. Some studies report conception rates comparable to conventional semen and successful production of about 74% female calves without compromising acrosomal integrity or sperm quality [25].

Differences in stress tolerance between X- and Y-bearing sperm have also been studied for sex manipulation. Y-bearing sperm are more vulnerable to environmental stressors, including pH changes, and acidic semen extenders can raise the proportion of X-bearing sperm in porcine semen [26]. Acidic conditions may impact sperm motility, metabolism, and swimming behavior [27, 28]. Therefore, adjusting extender pH could be a practical, farm-level method to increase the number of female offspring in pigs [26, 29] and goats [30].

Microfluidic chip–based separation is an advanced, low-cost technique that can be integrated with other sex-sorting methods. Devices using electrical fields, such as dielectrophoretic, have achieved about 70% *in vitro* accuracy [31, 32]. When combined with antibodies or magnetic beads, microfluidic sorting efficiency can reach around 80% [8]. Magnetic-activated cell sorting (MACS), which employs antibody-conjugated magnetic microspheres to target sex-specific antigens [33], provides high precision and causes minimal damage to sperm cells. However, challenges remain in large-scale purification where target-specific antibodies are unavailable [18, 34, 35]. MACS is beneficial because it is cost-effective, produces high yields of sexed sperm, and maintains good post-thaw quality, although its overall accuracy still needs further validation [18, 21, 25].

Despite significant progress in sperm sex-sorting technologies over the past 20 years, important knowledge gaps still exist across different species, methods, and real-world applications. Most research primarily focuses on flow cytometry, which limits understanding of how well newer methods, such as immunological sorting, centrifugation, microfluidics, and magnetic bead–based separation perform in practical settings. Additionally, most studies are conducted *in vitro*, with few *in vivo* or field trials that validate pregnancy outcomes, fertility rates, or offspring sex ratios for these alternative techniques. This makes it difficult to compare technologies systematically or develop standardized protocols for commercial and farm use. There is also a notable geographical imbalance; North America mainly researches flow cytometry, while Asia produces more work on lower-cost alternatives, but comparative global data are rare. Existing studies vary greatly in methods, reporting, and sample sizes, reducing the reliability of cross-study comparisons. Importantly, no comprehensive multi-species review has assessed the overall effectiveness of sex-sorting technologies or summarized their reproductive results with strong meta-analyses. Similarly, the role of these technologies in promoting sustainability, ethical livestock practices, and One Health approaches is still underexplored. These gaps emphasize the need for an updated, comprehensive global review of sperm sex-sorting over the last two decades.

The current study aims to fill these gaps through a comprehensive **systematic review and meta-analysis** of sperm sex-sorting research published between 2005 and 2025. Specifically, it seeks to (1) critically examine the global distribution, methodological variety, and technological progress of sperm sex-sorting techniques across different animal species; (2) quantitatively compare the **effectiveness of various methods**, especially flow cytometry and centrifugation, based on pregnancy rates and offspring sex ratios in *in vivo* studies; (3) summarize trends in *in vitro* performance metrics, including separation efficiency, sperm motility, and viability; (4) evaluate heterogeneity, methodological quality, and publication bias among studies; and (5) identify emerging innovations and unresolved challenges to guide future research, technological development, and commercial use. Ultimately, this research aims to provide the first multi-species, worldwide synthesis of sex-sorting technologies, clarifying their practical effectiveness and establishing priorities for research and development that support sustainable livestock production, animal welfare, and long-term One Health benefits.

## MATERIALS AND METHODS

### Ethical approval

Ethical approval was not required for this systematic review because it involved the analysis of previously published studies and did not include any direct experimentation on animals or humans. All data extracted were obtained from publicly accessible peer-reviewed literature, and no identifiable or confidential information was used.

### Study period

The study was conducted from June to August 2025. The data were extracted, analyzed and interpreted at Animal Innovation Research Group, Akkhraratchakumari Veterinary College, Walailak University, Nakhon Si Thammarat 80160, Thailand.

### Study design

This systematic review was conducted following the Preferred Reporting Items for Systematic Reviews and Meta-Analyses (PRISMA) 2020 guidelines [36] (Supplementary File, Tables S1 and S2). All methodological steps were predefined and rigorously followed to ensure transparency, reproducibility, and adherence to systematic review standards.

### PICOS framework

This systematic review was structured using the PICOS framework to clearly define the research question, inclusion criteria, and analytical outcomes.

- Population (P): domestic and wild animal species where sperm sex-sorting technologies were applied, including livestock (cattle, buffalo, pigs, sheep, goats), companion animals (dogs, cats, horses, donkeys), and wildlife.
- Intervention (I): any sperm sex-sorting technique used to separate X- and Y-bearing sperm, such as flow cytometry, centrifugation-based methods, immunological sorting, magnetic bead separation, modified buffer systems, and microfluidic devices.
- Comparator (C): comparisons among sex-sorting techniques or subgroups within the same method; however, conventional unsorted semen was not consistently available as a control.
- Outcomes (O): primary outcomes included pregnancy rate and proportion of female offspring from *in vivo* studies; secondary outcomes included *in vitro* parameters such as separation efficiency, sperm motility, and viability.
- Study design (S): original experimental studies (*in vitro* or *in vivo*) published in English between 2005 and 2025. This structured framework guided the development of eligibility criteria, data extraction, and synthesis strategy.

### Search strategy

A comprehensive literature search was initially conducted in PubMed, followed by supplementary searches in Scopus and Google Scholar. Boolean operators (“AND,” “OR”) were used to refine the results. Search terms included: “sperm sexing” OR “sperm sorting” OR “sex-sorted semen” OR “X-sperm” OR “Y-sperm” AND (“livestock” OR “animals”) AND (2005:2025[dp]). The search period spanned from January 2005 to July 2025, with the final search completed on July 31, 2025.

### Eligibility criteria and study selection

Study selection consisted of three stages: (1) screening titles, (2) screening abstracts, and (3) evaluating full texts.

### Inclusion criteria

- Published between 2005 and 2025
- Original research articles
- Reported effectiveness of sperm sex-sorting technologies
- Written in English
- Full-text available

### Exclusion criteria

- Review articles, conference papers, books
- Non-English articles without an accessible full-text
- Studies lacking sex-sorting outcome data

A total of 91 studies were included in the systematic review.

### Review of eligible studies

After selecting the eligible articles, each study underwent a thorough full-text review to verify methodological rigor, relevance, and appropriateness for inclusion. Two independent reviewers evaluated each study, examining objectives, clarity of methodological reporting, sex-sorting techniques used, species and sample details, and the availability of extractable outcome data (such as separation efficiency, sperm quality parameters, pregnancy rate, and female-offspring proportion). Studies missing essential methodological details, showing ambiguous or incomplete sex-sorting results, or lacking usable numerical data were excluded at this stage. For the remaining studies, reviewers confirmed the presence of well-defined experimental groups, clearly identified interventions, and outcome measures aligned with the predefined PICO/PICOS criteria. Any disagreements or uncertainties encountered during evaluation were resolved through discussion until consensus was achieved. This systematic, independent review ensured only high-quality, data-rich studies were included in the final systematic review and meta-analysis.

### Data extraction

Data from each eligible article were independently extracted and entered into Google Sheets (Google, California, USA). The complete dataset is accessible in the Open Science Framework repository (https://osf.io/ew8kb/files/2r6m5). Extracted variables included:

- Author name and year
- Animal species and country
- Sex-sorting method
- Study type (*in vitro*/*in vivo*)
- Separation efficiency
- Sperm motility and viability
- Pregnancy rate
- Percentage of female offspring
- Quality assessment scores

All extracted data and meta-analysis codes are included in the supplementary materials or can be requested.

### Descriptive analysis

Descriptive statistics summarized the distribution of studies across species, countries, and technologies. For datasets unsuitable for meta-analysis, mean, median, and standard deviation were calculated. Only outcomes reported in at least three independent studies (e.g., separation efficiency, motility, viability) were included in descriptive analyses.

### Meta-analysis

A minimum of three *in vivo* studies was required for each outcome. A **random-effects proportional meta-analysis** was used to account for variation between studies caused by differences in species, methodologies, and experimental conditions. Primary pooled outcomes included:

- Pregnancy rate, expressed as % of pregnant animals
- Female offspring proportion, expressed as % of total offspring

Proportions were adjusted and weighted based on sample sizes. Subgroup analyses were performed when enough studies were available, especially for flow cytometry–sorted semen.

### Assessment of heterogeneity

Heterogeneity was evaluated using:

- I² statistic (<50% low, 50–75% moderate, >75% high)
- Tau (T) and Tau² (T²)
- H²

Forest plots were visually inspected to evaluate the consistency of effect sizes across studies.

### Assessment of publication bias

Two reviewers (TY and TW) extracted and verified data, while a third reviewer (OH) resolved disagreements. Study quality was assessed using the Joanna Briggs Institute checklist for observational studies [37]. Risk of bias was evaluated across:

- Source reliability
- Methodological quality
- Reporting transparency

Publication bias was evaluated through funnel plots, Egger’s test, and Begg’s test, with p < 0.05 signifying potential bias.

### Statistical analysis

Descriptive statistics were calculated using Google Sheets. Meta-analyses, heterogeneity assessments, and publication bias tests were conducted with **Jamovi (version 2.6.13)** [38] utilizing the meta-analysis module, and **MedCalc (version 23.3.4)** (MedCalc Software Ltd., Ostend, Belgium). A significance level of **p < 0.05** was used.

## RESULTS

### Study selection

**Figure 1:**
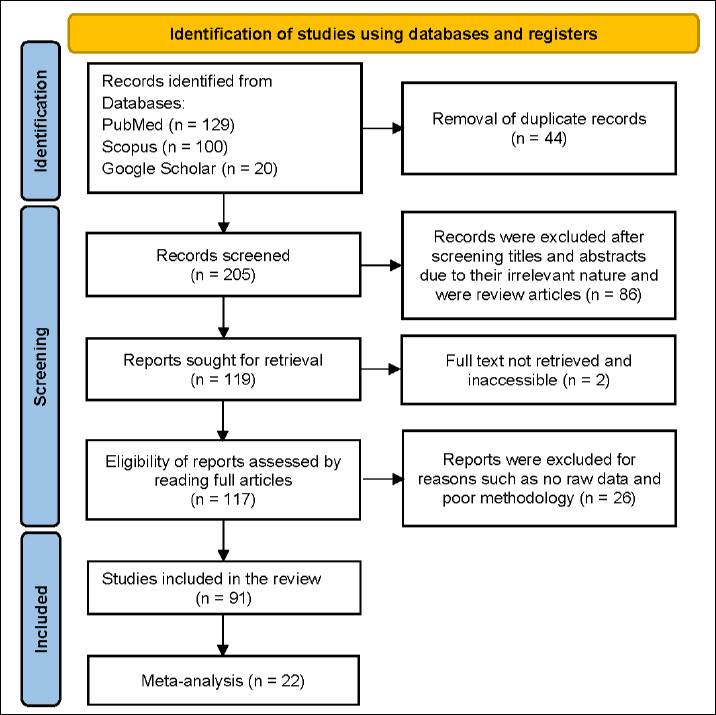
PRISMA flow diagram depicting the study selection process in this review.

The study selection process followed the PRISMA flow diagram (Figure 1). A total of 249 records were identified through electronic searches of PubMed, Scopus, and Google Scholar. After removing 44 duplicates, 205 records remained for screening. Title and abstract screening led to the exclusion of 114 studies based on the predefined inclusion and exclusion criteria. Full-text assessment was conducted for 117 articles, of which 91 met all eligibility criteria and were included in the systematic review. Of these, 22 studies provided enough quantitative data for inclusion in the meta-analysis evaluating pregnancy rates and the proportion of female offspring. Details of all studies included in both the systematic review and meta-analysis are provided in Supplementary Tables S4–S7.

### Characteristics of included studies

#### Sex-sorting techniques

Out of the 91 eligible studies, sperm sex-sorting methods were grouped into six main categories. Flow cytometry was the most commonly examined method (64 articles), followed by antibody-based methods (7 articles), centrifugation (7 articles), magnetic bead separation (5 articles), modified buffer systems (5 articles), and microfluidics-based techniques (3 articles).

#### Geographical distribution

The research covered 18 countries across four continents (Table 1). Asia contributed the most publications (32 articles), followed by Europe (25), North America (17), and South America (9). The United States led with 17 articles, followed by China with 10, and Spain and Thailand each with 9. In Asia, antibody-based techniques (11 articles) and magnetic bead–assisted separation (4 articles) were most common, while nearly all North American research focused on flow cytometry (24 of 25 studies).

**Table 1:** Countries and continents where sperm technology was performed in the last 20 years.

Continent	Country	Publication for each country	Publication for all continents
Asia	China	10	32
	Thailand	9	
	Korea	7	
	Indonesia	2	
	Japan	2	
	Pakistan	1	
	India	1	
North America	USA	17	17
South America	Brazil	6	9
	Argentina	2	
	Mexico	1	
Europe	Spain	10	25
	Italy	7	
	Ireland	2	
	Germany	4	
	Bulgaria	1	
	Switzerland	1	
Australia	Australia	8	8

#### Study settings and species

Of the included studies, 48 were conducted *in vitro*, 31 *in vivo*, and 10 included both experimental components. Across all publications, sperm sex-sorting was performed in 13 species. Cattle were the most frequently studied (49 articles), followed by pigs (11), buffaloes (5), and deer (5). Livestock species made up 71 studies, while companion animals (dogs, cats, horses, donkeys) appeared in 9 studies. Wildlife species, including rhinoceros, deer, and dolphins, were featured in 10 publications, and one study used mice as a laboratory model.

### Publication trends and risk of bias

An overview of the publication timeline and risk of bias is provided in Supplementary Table S4. Research on sperm sex-sorting has steadily increased over the past two decades.

- 2005–2010: Early research focused on flow cytometry and low-dose insemination in cattle, buffalo, deer, and pigs, mostly involving studies with low risk of bias.
- 2011–2015: Publication rates rose, highlighting *in vitro* embryo production, sperm quality assessment, and the development of alternative sorting methods, including both low- and moderate-risk studies.
- 2016–2020: Studies expanded to include new methods such as microfluidics, magnetic nanoparticles, and antibody-based sorting, reflecting increasing technological innovation.
- 2021–2025: This period saw the highest number of publications, including experimental techniques with medium to high risk of bias due to early developmental stages.

Overall, publication patterns demonstrate increasing global engagement, diversification of technologies, and an ongoing need for methodological refinement.

### *In vitro* findings

Only two sex-sorting techniques, flow cytometry (38 articles) and microfluidics (3 articles), provided sufficient data for *in vitro* descriptive analysis.

- Sorting efficiency:
  Flow cytometry (8 studies): mean 73.51% ± 34.41%, median 90%
  Magnetic beads (3 studies): mean 83.64% ± 5.58%, median 81.43%
- Sperm motility:
  Centrifugation (4 studies): mean 66.02% ± 29.19%, median 78.16%
  Flow cytometry (20 studies): mean 54.43% ± 26.06%, median 50.75%
  Microfluidics (2 studies): mean 43.5% ± 12.02%
- Sperm viability:
  Flow cytometry (12 studies): mean 65.41% ± 25.82%, median 74%
  Centrifugation (3 studies): mean 78.50% ± 12.12%, median 78.25%

Due to high variability in methods and reporting, meta-analysis for *in vitro* parameters was not feasible.

### *In vivo* findings

Among the 31 *in vivo* studies, flow cytometry was used most frequently (25 articles), followed by centrifugation-based sorting (3), with one study each using antibody-based separation, magnetic bead–assisted techniques, and modified buffers. Only data from flow cytometry and centrifugation were sufficient for meta-analysis.

### Meta-analysis: Pregnancy rate

A total of 21 studies were included in the pregnancy rate meta-analysis (Table S5), covering five species: cattle (10 studies, 2,391 animals), buffalo (3 studies, 4,769 animals), deer (3 studies, 285 animals), sheep (1 study, 56 animals), and pigs (1 study, 57 animals) (Table 2).

**Table 2:** Pregnancy rate after artificial insemination with sex-sorted semen

Animals	Number of published articles	Sex-sorting technique	Number of animals	Pooled estimated			Heterogeneity		

				% Pregnancy	95% CI	p-value	H^2^	I^2^	T^2^
All species	18	Flow	7558	46.47	39.74-53.20	<0.001	24.19	95.86	0.01
Cattle	10	Flow	2391	37.67	27.55-47.78	<0.001	26.35	96.2	0.02
Buffalo	3	Flow	4769	53.33	45.14-61.52	<0.001	4.85	79.41	0.004
Deer	3	Flow	285	47.47	41.29-53.66	<0.001	1.04	4.47	0.0002
All species	3	Centrifuge	210	66.49	28.68-100.04	<0.001	34.45	97.09	0.10

CI = Confidence interval

- Overall pooled pregnancy rate (all species): 46.47% (p < 0.001)
- Species-specific rates:
  Cattle: 37.67%
  Buffalo: 53.33%
  Deer: 47.47%

### Heterogeneity

Flow cytometry studies exhibited very high heterogeneity:

- All species: I² = 95.86%, H² = 24.19
- Cattle: I² = 96.20%, H² = 26.35
- Buffalo: I² = 79.41%, H² = 4.85
- Deer: I² = 4.47%, H² = 1.04 (indicating highly consistent results)

**Figure 2:**
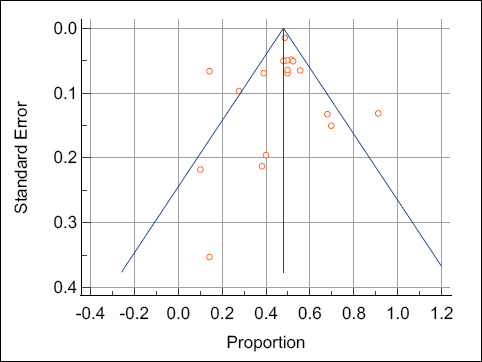
Funnel plot illustrating the meta-analysis of pregnancy rates in animals following artificial insemination with flow cytometry-based sex-sorted semen.

Funnel plot inspection revealed no major asymmetry (Figure 2), and both Egger’s and Begg’s tests were nonsignificant (P > 0.05).

### Centrifugation-based sex-sorting

Three studies (all in cattle) were included:

- Pooled pregnancy rate: 66.49% (p < 0.001)
- Heterogeneity: I² = 97.09%
- H² = 34.45

Egger’s and Begg’s tests indicated no publication bias (p > 0.05).

**Figure 3:**
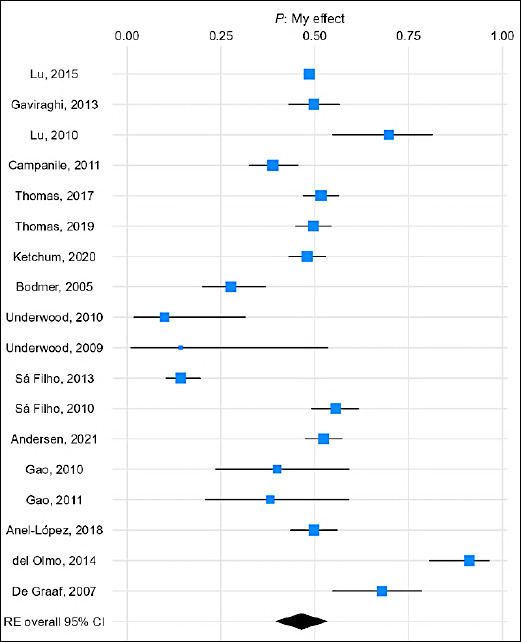
Forest plot showing the meta-analysis of pregnancy rates in animals following artificial insemination with flow cytometry-based sex-sorted semen.

Forest plots (Figure 3) and species-specific subgroup analyses (Figures S1–S5) further demonstrate these findings.

### Meta-analysis: Female offspring percentage

Eleven flow cytometry studies across five species (cattle, buffalo, deer, pigs, dolphins) reported offspring sex-ratio data (n = 2,627 offspring) (Table 3).

**Table 3:** Estimated female offspring after artificial insemination with sex-sorted semen obtained by flow cytometry.

Animals	Number of published articles	Number of animals	Pooled estimated			Heterogeneity		

			% Female offspring	95% CI	p-value	H^2^	I^2^	T^2^
All	11	2627	81.72	74.43-89.00	< 0.001	12.91	92.25	0.01
Cattle	5	361	84.4	73.28-95.53	< 0.001	9.79	89.79	0.01

CI = Confidence interval

- Overall pooled female offspring proportion: 81.72%
- Cattle-only subgroup (5 studies, 361 offspring): 84.40% (p < 0.001)

### Heterogeneity

- All species: I² = 92.25%, H² = 12.91
- Cattle subgroup: I² = 89.79%, H² = 9.79

**Figure 4:**
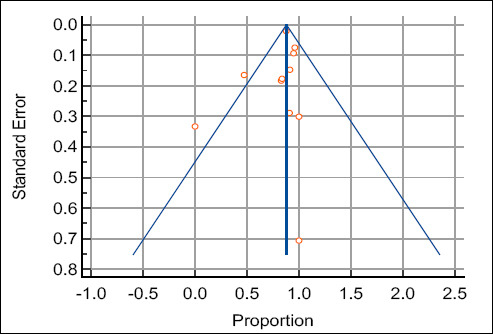
Funnel plot illustrating the meta-analysis of the proportion of female offspring following artificial insemination with flow cytometry-based sex-sorted semen.

Funnel plot symmetry and statistical tests (Egger’s p = 0.48; Begg’s p = 0.39) indicated no publication bias (Figure 4).

**Figure 5:**
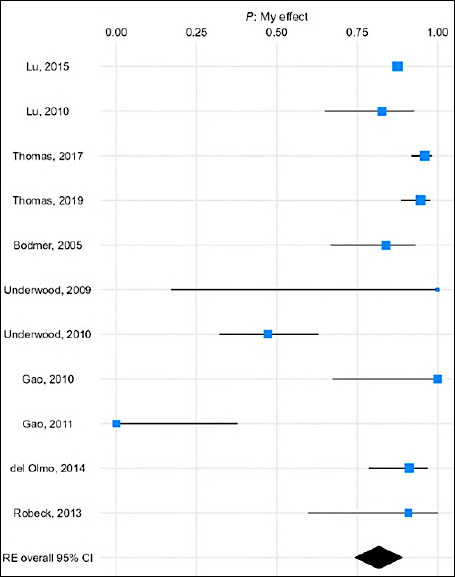
Forest plot showing the meta-analysis of the proportion of female offspring following artificial insemination with flow cytometry-based sex-sorted semen.

The forest plot (Figure 5) showed effect sizes ranging from 0.20 to 0.90, with most clustered between 0.60 and 0.80. The random-effects model demonstrated the strong overall effectiveness of sex-sorting technologies in achieving female-biased outcomes.

### Commercialization status

Commercial flow-cytometry–sorted semen (SexedULTRA®, **STgenetics**, USA) was reported in four *in vivo* studies [14, 15, 39, 40]. To date, no commercial sex-sorting technologies (such as magnetic beads, microfluidics, or immunological sorting) have been used in validated *in vivo* trials.

## DISCUSSION

### Advantages and limitations of sperm sex-sorting technologies

Table 4 summarizes the advantages and disadvantages of each sperm sex-sorting method. Flow cytometry is the most accurate and well-validated method, routinely delivering over 90% separation efficiency and more than 70% female offspring. However, despite its accuracy, this technique requires expensive equipment, specialized skills, and complex laboratory infrastructure, which restricts its use in resource-limited areas.

**Table 4:** Proposed summary of the advantages and disadvantages of different sex-sorted sperm technologies.

Technology	Advantages	Disadvantages
Flow cytometry	•Most accurate and widely validated method. •>90% separation efficiency *in vitro*. •Commercially available. •High number of studies •Applicable to multiple species. •Produces highly predicable offspring •>70% female offspring.	•High equipment, maintenance, and operation costs. •Requires skilled personnel and sophisticated laboratory equipment •Reduces sperm viability and motility induced by laser exposure and mechanical stress. •Limited accessibility in developing countries
Centrifugation (Percoll, albumin gradient, or differential centrifugation)	•Relatively inexpensive and simple to perform. •Requires minimal equipment and expertise. •Less mechanical damage compared with flow cytometry. •Moderate number of studies	•Low to moderate accuracy, <70%. •Limited *in vivo* studies, but higher than other alternatives •Sperm damage from repeated centrifugation. •Not yet commercially standardized.
Special buffer (e.g., pH, charge, or protein-based separation)	•The most non-invasive and low-cost approach. •It is the easiest way to implement under laboratory or farm conditions. •Minimal impact on sperm structure and function.	•Low to moderate accuracy, <70%. •Limited *in vivo* studies •Not yet commercially standardized.
Magnetic Bead–Based separation (with or without antibody conjugation)	•Promising *in vitro* results, > 80% separation efficiency when using specific antibodies. •Non-invasive and less mechanical stress on sperm cells.	•Limited *in vivo* studies •Expensive reagents compared with other alternatives •Risk of nonspecific binding or sperm damage when using an antibody-based antibody •Not yet commercially standardized
Microfluidic chips (with or without an electric field)	•Gentle, non-invasive, (without electricity) •Potential for automation, miniaturization, and on-farm applications. •Low sample requirement and minimal reagent use. •Cheaper than flow cytometry	•Moderate accuracy around 60%-70% •No *in vivo* study •Lower throughput compared with flow cytometry. •High-tech equipment is required to produce chips. •Lowest number of studies •No commercialization

Centrifugation-based methods offer a simpler, more cost-effective solution with less sperm damage and minimal infrastructure needs. Nonetheless, they deliver moderate accuracy (under 70%), face standardization issues, and show limited consistency in *in vivo* validation. Special buffer systems are minimally invasive and suitable for basic lab or field settings, but they often lack reproducibility. Magnetic bead separation can reach high *in vitro* efficiency (over 80%) if selective antibodies are available, though it remains experimental and somewhat costly. Microfluidic platforms provide gentle handling, low-cost, and potential for automation, but currently achieve moderate accuracy (60%–70%) and lack validated *in vivo* data or commercial use. Overall, flow cytometry remains the gold standard, while magnetic beads and microfluidics are promising emerging technologies that need further development.

### Global research patterns and technological adoption

This review identified flow cytometry as the most frequently used method for sex-sorting, with cattle as the primary species studied. To the authors’ knowledge, this is the first systematic meta-analysis comparing pregnancy outcomes across various sex-sorting technologies and species. The dominance of flow cytometry research in the United States reflects the country’s robust dairy and beef industries and its well-established commercial sex-sorting sector [41, 42].

Unlike earlier reviews that mainly focused on Western livestock systems, the present study includes significant evidence from Asia, highlighting emerging innovation in affordable, alternative sex-sorting methods. These regional trends indicate a rising international demand for accessible technologies suited to diverse livestock systems.

### Pregnancy outcomes across technologies and species

Only flow cytometry and centrifugation provided enough data for a meta-analysis of pregnancy outcomes. The overall estimated pregnancy rate using flow cytometry–sorted semen was 46%, although cattle showed lower rates than buffalo and deer. Pregnancy outcomes are affected by complex factors, such as species, breed, season, nutrition, semen quality, AI strategy (natural or synchronized estrus), and animal age [43, 44], making direct cross-species comparisons difficult.

Previous meta-analyses have shown that flow cytometry–sorted semen results in lower pregnancy rates (41%) compared to conventional semen (55%), with dairy cows having lower success rates than beef cows [41]. In contrast, the current analysis indicated that centrifugation-sorted semen achieved a higher estimated pregnancy rate of 67%, though this is based on only three studies and should be interpreted with caution. Nonetheless, sex-sorted semen remains economically beneficial, as reproductive efficiency improves when daily pregnancy rates exceed 25% [45].

In buffalo, typical pregnancy rates with conventional semen range from 30% to 50% [46], while this review showed flow cytometry–sorted semen achieving over 50%. Differences among breeds (such as Murrah versus Nili-Ravi bulls), seasons, and insemination technicians may also influence these rates [47].

### Female offspring proportion and deviations from expected rates

Flow cytometry-based sex-sorting was the sole method with enough publications to analyze the offspring sex-ratio. The combined proportion of female offspring was 82% across species, which is below the typically reported figure (>90%) [10, 48].

This discrepancy may result from several biological and technical factors:

- Sex-sorted semen is not perfectly purified.
- Fertilization depends on a single sperm, while millions enter the oviduct.
- Variation in the oviductal microenvironment may differentially favor X- or Y-bearing sperm.
- Under certain physiological conditions, Y-bearing sperm may have a competitive advantage [49]

These dynamics might explain why field results sometimes do not meet laboratory standards.

### Economic and practical considerations in adopting sex-sorting technologies

Although flow cytometry remains costly, increased availability and decreasing prices continue to make it more accessible. Several cost–benefit studies have shown higher profitability through increased female calf production, better genetic progress, and successful crossbreeding results [50]. Using X-sperm can also lower dystocia and stillbirth rates, as male calves tend to cause more birthing complications [51]. When combined with estrus synchronization, sex-sorted semen further improves reproductive success [52]. Japan has reported reductions in production costs, better milk quality, and higher herd productivity through the use of sex-sorted semen [53].

### Advances and research momentum in Asia

This review emphasizes notable research efforts across Asia, especially in developing alternative sperm sex-sorting methods such as antibody-based sorting, magnetic bead separation, microfluidics, centrifugation, and modified buffer techniques. This pattern may indicate strong demand for sex-sorted semen in a region with limited access to commercial flow cytometry. Since Asia accounts for approximately 60% of the world’s population [54] and hosts one of the largest livestock industries [55], there is a significant interest in affordable technologies.

### Progress and limitations of alternative sex-sorting methods

Although many alternative methods have been developed, most research remains limited to *in vitro* testing, with few *in vivo* studies. Magnetic bead–based separation, whether with antibody conjugation or not, has shown promising results, achieving over 80% efficiency in three studies, but these findings come from only two research groups [18, 21, 56].

Currently, only centrifugation-based sex-sorting has sufficient *in vivo* publications to allow for meta-analysis, although data on *in vitro* confirmation of X/Y proportions are still limited. Overall, these results highlight the necessity for more *in vivo* studies to validate the practical use of emerging technologies.

### Applications beyond livestock: Companion animals and wildlife

Although most research focuses on livestock, interest is increasing in using sex-sorting technologies for companion animals and wildlife. In horses, sex-sorting helps improve breeding strategies, especially in industries where gender preference affects value. Additionally, companion animals are used as models to develop assisted reproduction techniques that can benefit endangered species [2, 57].

In wildlife research, emerging studies involving dolphins and deer indicate potential uses in conservation, especially for endangered species with skewed sex ratios or low reproductive rates [58]. This is an important but underexplored area in sex-sorting research.

### Study limitations

#### High heterogeneity

Most meta-analyses displayed high heterogeneity (I² > 95%). Studies employing centrifugation exhibited more variability than those using flow cytometry, likely due to differences in genetics, management, environment, or lab protocols. Deer studies utilizing flow cytometry showed very low heterogeneity, indicating consistent results, possibly because of a limited number of research groups and similar sample sources. Variations in male influences on sperm separation and sex-ratio biases have also been documented [59].

#### Language bias

Only English-language publications were included, which could introduce language bias. Non-English studies might offer additional data but often lack detailed methodology or have variable peer-review quality. English-language studies were favored for reliability and reproducibility, though this may underrepresent research from non-English-speaking regions.

#### Lack of control groups

A significant limitation was the lack of paired unsorted (conventional) semen data in many studies, which hindered direct comparison between sex-sorted and conventional semen. Future reviews should focus on studies that include both semen types to allow for more comprehensive assessments of their relative effectiveness and reproductive outcomes.

## CONCLUSION

This systematic review and meta-analysis summarized two decades of global research on sperm sex-sorting technologies, highlighting significant advances in both established and new methods. Flow cytometry remained the most accurate and extensively validated approach, achieving over 90% sorting efficiency and an average of 82% female offspring. Its overall pregnancy rate across species was moderate at 46%, with higher success observed in buffalo and deer compared to cattle. Centrifugation-based sorting, though less studied, showed promising results with a 67% pregnancy rate, indicating its potential as a cost-effective, field-friendly alternative. *In vitro* tests suggested that magnetic bead–based methods and centrifugation could improve sperm motility or viability in certain circumstances, but these results need further confirmation.

Practically, the findings demonstrate that sex-sorting offers significant reproductive and economic advantages, especially in dairy, buffalo, and conservation efforts. Flow cytometry–based semen continues to be beneficial for producing replacement heifers, reducing issues like dystocia in male calves, boosting genetic improvement, and increasing herd productivity over the long-term. New methods such as microfluidics, antibody-based sorting, and magnetic bead separation provide affordable, accessible alternatives for areas lacking commercial flow cytometry services and could broaden the use of sex-sorting in wildlife, pets, and small-scale farming.

This study’s strengths include a thorough 20-year global evidence review, evaluation across multiple species, the first meta-analysis comparing flow cytometry and centrifugation outcomes, and strict adherence to PRISMA guidelines. Yet, it has limitations such as high heterogeneity (I² > 90%) due to differences in species, breeds, AI methods, and lab protocols; scarce in vivo data for alternative techniques; possible language bias from only including English articles; and a lack of sufficient comparative data with conventional (unsorted) semen, which hinders direct comparison of reproductive performance.

Future research should focus on conducting large-scale, standardized *in vivo* trials for emerging technologies, harmonizing *in vitro* assessment methods across studies, and performing multi-country evaluations to better understand environmental and genetic factors affecting success rates. Furthermore, it is important to include studies that compare sex-sorted and conventional semen simultaneously, to strengthen economic and fertility analyses. Extending research into wildlife and conservation efforts could also be valuable for tackling sex-ratio imbalances and aiding endangered species initiatives.

In conclusion, while flow cytometry remains the gold standard for precise and consistent sperm sex-sorting, other technologies show promising potential to improve accessibility, lower costs, and broaden applications across various species. Ongoing innovation, thorough validation, and expanded research efforts will be essential for making sex-sorting techniques more practical, globally adaptable, and sustainable.

## DATA AVAILABILITY

The data supporting this study’s funding are available from the Open Science Framework (OSF) (https://osf.io/ew8kb/files/2r6m5).

## AUTHORS’ CONTRIBUTIONS

TY: Conception, experimental design, data collection, data analysis, initial draft writing, final draft review and revision, and funding acquisition. OH: Conception, supervision, and final draft rewriting. TW: Conception, experimental design, data collection, data analysis, original draft writing, final draft review and rewrite, project administration, and funding acquisition.
